# Partial Splenic Embolization for Portal Hypertension Before Radical Nephrectomy in Renal Cell Carcinoma Patient: A Case Report

**DOI:** 10.1002/iju5.70108

**Published:** 2025-10-13

**Authors:** Shunsuke Watanabe, Keita Tamura, Kyohei Watanabe, Yuto Matsushita, Hiromitsu Watanabe, Daisuke Motoyama, Teruo Inamoto

**Affiliations:** ^1^ Department of Urology Hamamatsu University School of Medicine Hamamatsu Japan; ^2^ Department of Developed Studies for Advanced Robotic Surgery Hamamatsu University School of Medicine Hamamatsu Japan

**Keywords:** nephrectomy, partial splenic embolization, portal hypertension, preoperative management, renal cell carcinoma

## Abstract

**Introduction:**

Partial splenic embolization is a valuable option to manage hypersplenism and reduce portal pressure while preserving splenic function. It may improve surgical safety in patients with portal hypertension.

**Case Presentation:**

A 67‐year‐old Japanese male with renal cell carcinoma and hepatitis B‐related liver cirrhosis presented with thrombocytopenia, collateral vessels, and a splenorenal shunt. Due to concerns regarding surgical risk, partial splenic embolization was performed preoperatively. Following the procedure, the platelet count increased, and collateral circulation decreased. These improvements enabled a safe left radical nephrectomy.

**Conclusion:**

Partial splenic embolization can be an effective preoperative strategy in patients with renal tumors and coexisting portal hypertension. By improving hematologic parameters and reducing vascular risk, it may facilitate curative surgery in high‐risk patients and expand treatment options.

**Summary**

- This report demonstrates that partial splenic embolization can effectively reduce portal hypertension and improve thrombocytopenia, enabling safer radical nephrectomy in renal cell carcinoma patients with cirrhosis.
- PSE may broaden surgical indications and improve treatment feasibility in high‐risk patients.

**Abbreviations**
CT: computed tomography
GEV: gastroesophageal varices
GR shunt: gastric renal shunt
IMV: inferior mesenteric vein
IVC: inferior vena cava
LGV: left gastric vein
LRV: left renal vein
MRI: magnetic resonance imaging
PLT: platelet count
PSE: partial splenic embolization
SGV: short gastric vein
SMV: superior mesenteric vein
SR shunt: splenorenal shunt
SV: splenic vein

## 1 Introduction

With advances in minimally invasive procedures such as laparoscopic and robot‐assisted surgeries, along with new drug developments, treatments for those with cancer have led to improved patient prognoses [1]. However, the growing availability of such treatments has also created new challenges related to tolerability [2]. Many cancer patients are elderly and have complications that make standard treatment difficult, such as cirrhosis. Cirrhosis can lead to portal hypertension, causing issues such as thrombocytopenia due to splenic hyperfunction, collateral vessel formation, and shunts, which may discourage surgical intervention.

Previously, splenectomy was used to manage splenic hyperfunction symptoms, but it is markedly invasive. Recently, partial splenic embolization (PSE) has been employed with positive outcomes to control these symptoms [3]. PSE, an interventional radiology procedure, reduces splenic volume by partially embolizing the splenic artery, which decreases splenic venous blood flow and portal pressure while preserving splenic function. Here, we report a case in which PSE facilitated safe nephrectomy in a patient considered high‐risk for surgery.

## 2 Case Presentation

**FIGURE 1:**
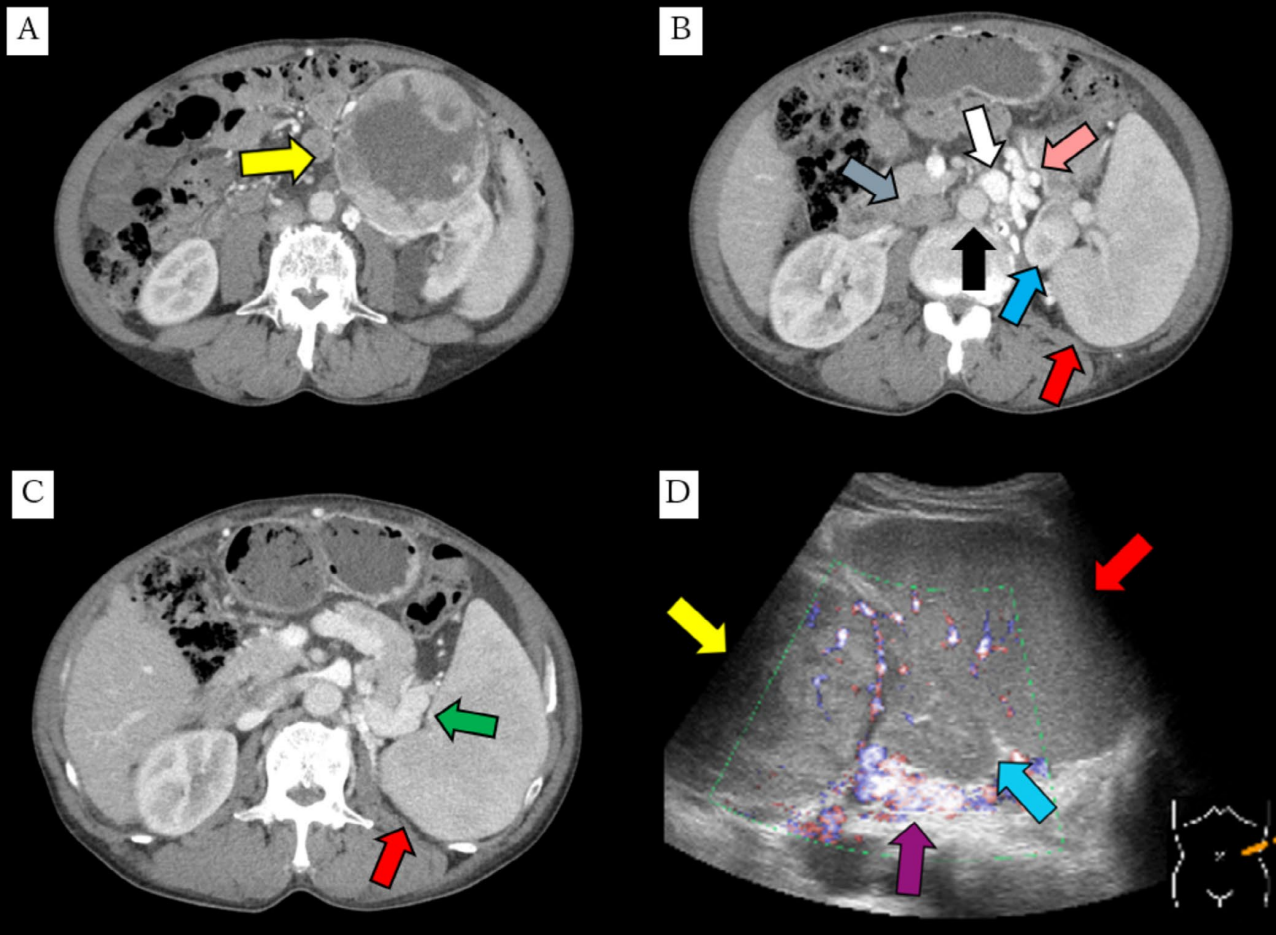
Contrast‐enhanced CT of the abdomen revealed a 10‐cm renal tumor (A) and well‐developed collateral blood vessels (pink arrow), enlarged spleen (red arrow), normal kidney (blue arrow), aorta (black arrow), inferior vena cava (gray arrow), renal vein (B), and dilated and tortuous splenic vein (green arrow) (C). Abdominal ultrasound showed a renal tumor (yellow arrow), normal kidney (blue arrow), enlarged spleen (red arrow), and splenorenal shunt (purple arrow) (D).

A 67‐year‐old male with liver cirrhosis and spinal canal stenosis presented to a local hospital with back pain, and subsequent MRI revealed an incidental left renal tumor. He was then referred to our hospital for further evaluation and treatment. Physical examination showed a distended yet soft abdomen with ascites. Contrast‐enhanced CT identified left renal cell carcinoma with well‐developed collateral vessels (Figure 1A–C), but no distant metastases, leading to renal cancer staging of cT2aN0M0. Abdominal ultrasound indicated a 10‐cm renal tumor, splenomegaly, and splenorenal shunt due to portal hypertension (Figure 1D). No gastroesophageal varices were observed on upper gastrointestinal endoscopy. Laboratory results were: WBC, 4.6 × 10^3^/μL; hemoglobin, 8.7 g/dL; platelets, 7.6 × 10^4^/μL; albumin, 2.5 g/dL; prothrombin time, 67% (INR, 1.16); total bilirubin, 1.0 mg/dL; Child–Pugh class B. Although nephrectomy was considered, we hesitated due to thrombocytopenia, collateral vessels, and the splenorenal shunt. Ultimately, we decided on preoperative PSE.

**FIGURE 2:**
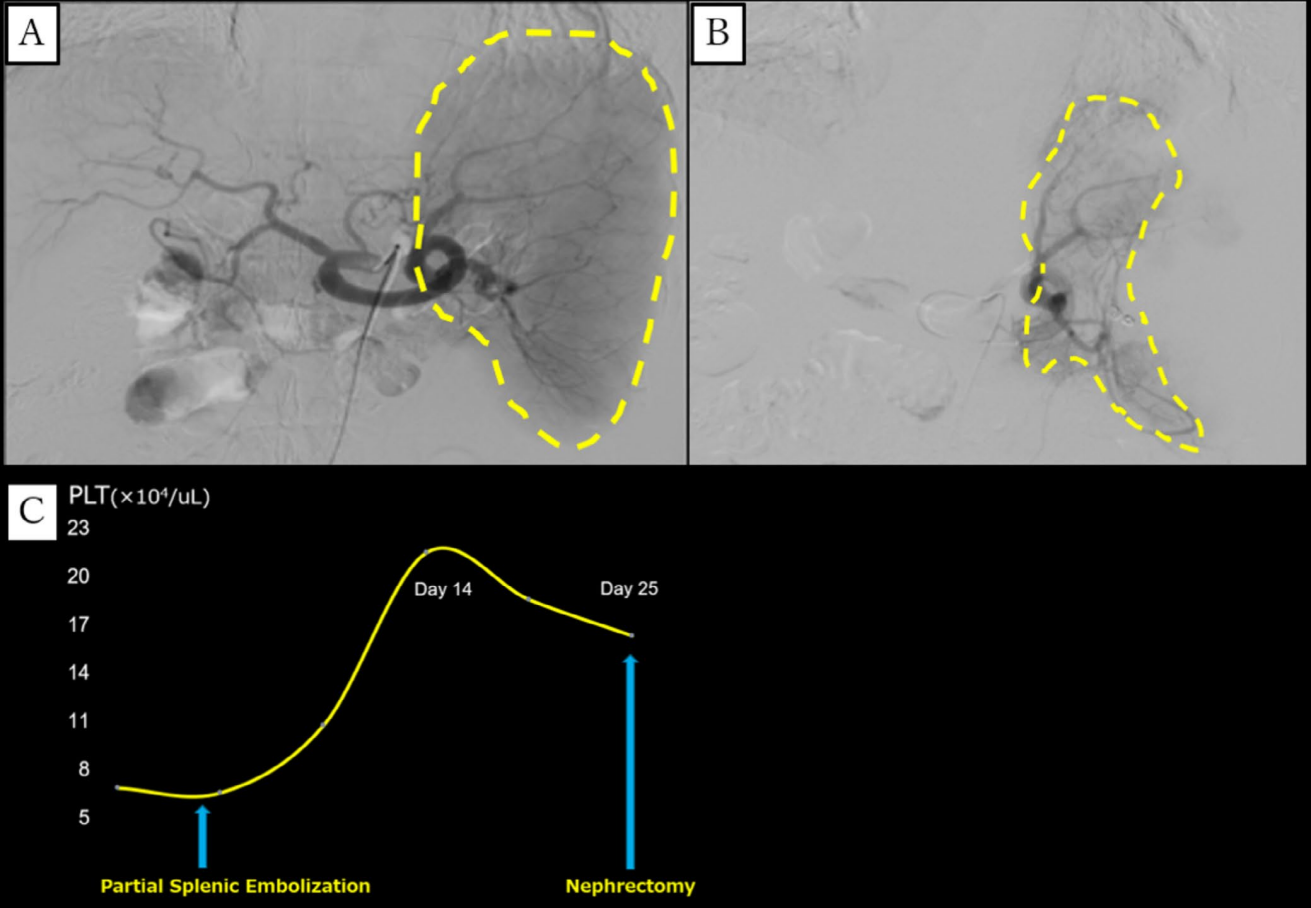
Celiac angiography before (A) and splenic artery angiography after (B) PSE, showing decreased splenic artery blood flow (yellow line). Platelet Count trends after PSE (C).

Through a femoral approach, a 5Fr catheter was inserted into the splenic artery via the right femoral artery using the Seldinger method. Splenic arteriography confirmed the arterial branches, and embolization was performed using a gelatin sponge with cephamedine and a coil to occlude approximately 80% of the splenic artery (Figure 2A,B). After PSE, the platelet count peaked at 22.0 × 10^4^/μL on Day 14 and then gradually declined. Radical nephrectomy was performed 25 days post‐PSE with platelets at 16.7 × 10^4^/μL (Figure 2C).

**FIGURE 3:**
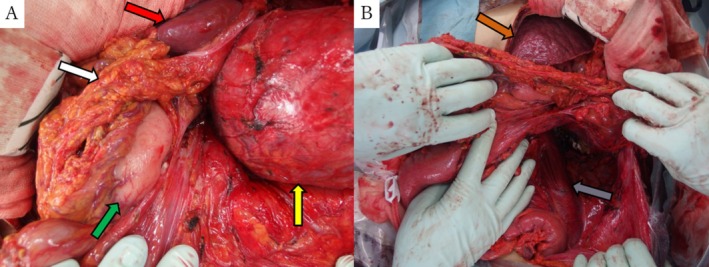
Renal tumor (yellow arrow), pancreas (white arrow), speckled spleen (red arrow), stomach (green arrow) (A), and nodular liver (orange arrow), psoas muscle (gray arrow) (B) were observed. The collateral blood vessels were not as prominent as had been predicted.

General and epidural anesthesia were administered, the patient was placed in a supine position, and the operation was started with upper and lower abdominal ventral midline celiotomy and a transperitoneal approach. During surgery, the spleen appeared partially infarcted and speckled, and perinephric collateral vessels were reduced (Figure 3A,B). The operation lasted 5 h and 8 min, with blood loss of 1700 mL (primarily ascitic fluid), and the patient received 840 mL of red blood cells and 240 mL of fresh frozen plasma. No platelet transfusions were performed. Hemoglobin rose from 8.6 g/dL pre‐ to 10.2 g/dL postoperatively. The pathological results were as follows: clear cell renal cell carcinoma 13.5 × 10 × 7 cm, G2 > 3 Fuhrman grade 4 INFb, pT2b, negative resection margin. The patient was discharged on postoperative Day 10 without major complications.

## 3 Discussion

Cirrhotic patients often exhibit abnormal coagulation, thrombocytopenia, and varices due to splenic hyperplasia, which complicates or even precludes surgery. Splenectomy has been a traditional treatment for hypersplenism, but carries high bleeding risks because of thrombocytopenia and coagulation abnormalities.

Splenic artery embolization was introduced in 1973 as an alternative to splenectomy [4], but initially had high complication rates. In 1979, PSE was developed with fewer complications, as it infarcts only part of the spleen, reducing blood flow while preserving function [5]. Compared with splenectomy, PSE shows similar efficacy with lower invasiveness [6] and adjustable infarction levels according to patient condition, with the option of repeat procedures if necessary [7].

Reported complications include fever, abdominal pain, pleural effusion, splenic abscess, venous thrombosis, and peritonitis [8]. These are generally less frequent than with splenectomy, and none occurred in the present case. Post‐PSE, platelet counts typically peak within 2–4 weeks before gradually declining [9], so the timing of PSE should be tailored to each patient's response. In our case, platelet counts were within the normal range preoperatively, and neither splenectomy nor platelet transfusion was required. Ultrasound by an experienced physician is crucial for PSE, while the treating radiologist should determine the optimal embolization rate by balancing hepatic reserve and complication risk. Although guidelines recommend 50%–70% infarction [10, 11, 12], we targeted 80% because the Child–Pugh score was not high and greater thrombocytosis was desired [12]. Ultrasound showed multiple collateral vessels initially, which decreased after PSE.

**FIGURE 4:**
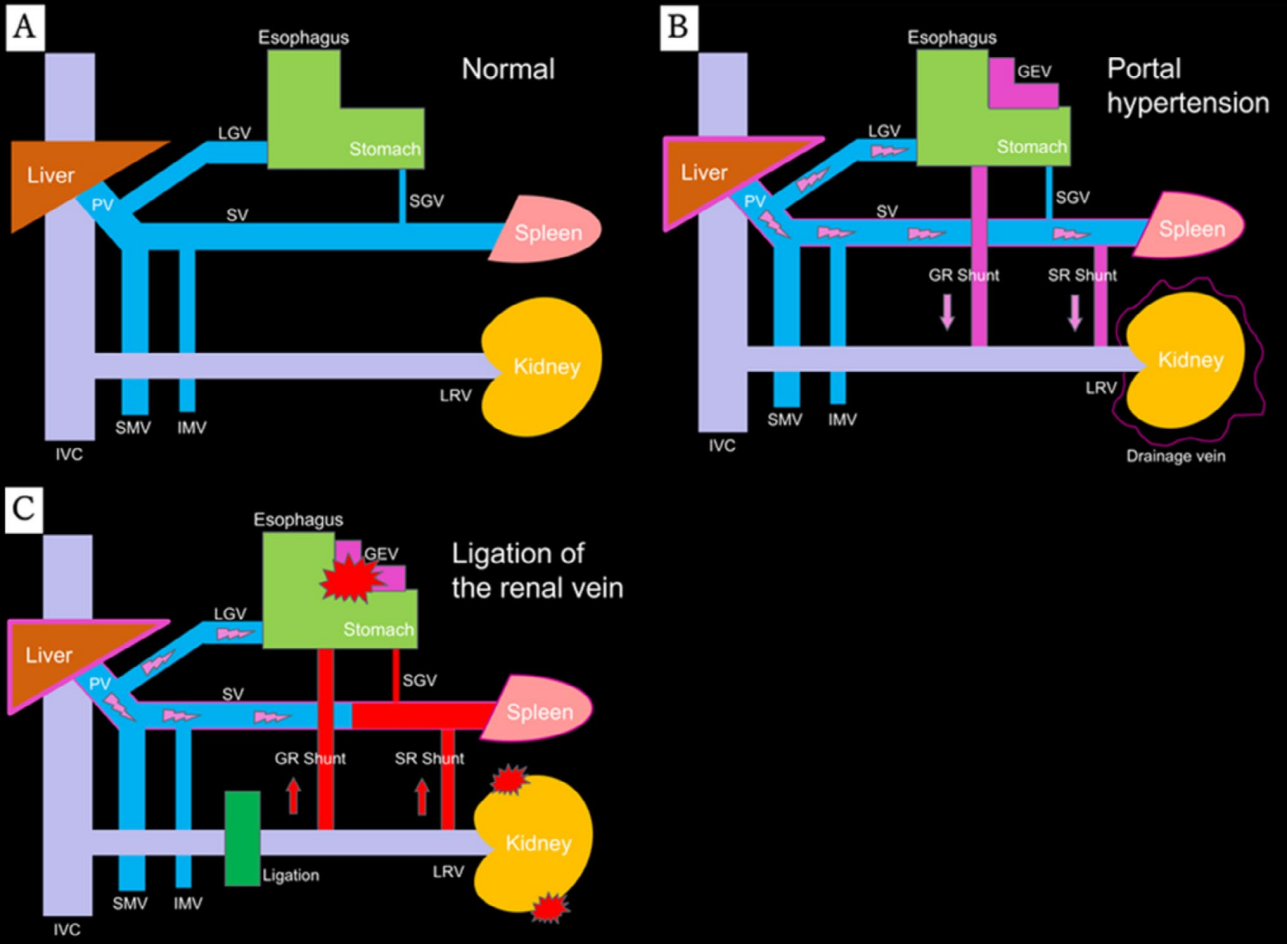
Relationship between inferior vena cava and portal systems normal (A), portal hypertension (B), ligation of renal vein (C).

PSE has been reported as a useful preoperative procedure in various surgeries [13, 14, 15] and may be particularly beneficial in renal cell carcinoma patients given the hemodynamic conditions. Normally, the renal veins drain into the inferior vena cava, while the splenic and left gastric veins form the portal vein (Figure 4A). In cirrhosis, portal hypertension produces gastrorenal and splenorenal shunts with perinephric collaterals [16] (Figure 4B). During nephrectomy, ligation of the renal vein may compress these collaterals and shunts, increasing bleeding risks and potentially rupturing gastroesophageal varices (Figure 4C). In Figure B, PSE decreases splenic artery blood flow and portal pressure, bringing the situation closer to that of Figure A. Poorly controlled portal hypertension may cause bleeding, as seen in Figure C. Thus, preoperative PSE can stabilize hemodynamics and lower complication risk. In this case, it improved platelet counts and reduced intraoperative bleeding.

In March 2023, thrombopoietin receptor agonists were approved in Japan for thrombocytopenia in chronic liver disease patients scheduled for invasive procedures. Our patient underwent surgery before this approval; thus, agonists were not used. Currently, their use with platelet transfusion may provide alternatives when PSE is considered too invasive.

Although platelet increases with PSE may be less than with splenectomy, PSE remains a practical option for patients unsuitable for invasive surgery. It improves perioperative safety, facilitates safer drug therapy, and expands treatment opportunities for cirrhotic patient [17, 18].

## 4 Conclusion

We performed PSE as a preoperative procedure for a renal cancer patient with cirrhosis, followed by successful nephrectomy. Beyond its preoperative role, PSE offers comparable control of portal hypertension to splenectomy with less invasiveness, making it a preferable option for high‐risk patients.

## Consent

Written informed consent for releasing this case report and accompanying images has been obtained from the patient.

## Conflicts of Interest

The authors declare no conflicts of interest.
